# Piperacillin-tazobactam resistance in *Klebsiella pneumoniae* is often associated with IS*26-*mediated *bla*_SHV-1_ amplification in a widespread *Klebsiella*-adapted plasmid

**DOI:** 10.1128/aac.01682-25

**Published:** 2026-03-24

**Authors:** Guilhem Royer, Maxime Danjean, Christophe Rodriguez, Vincent Fihman, Emmanuelle Gallois, Eve Tessier, Florence Reibel, Nicolas Cabanel, Philippe Glaser, Paul-Louis Woerther, Hervé Jacquier

**Affiliations:** 1 Unité de Bactériologie, Département de Prévention, Diagnostic et Traitement des Infections, Hôpital Henri Mondor, APHP, Créteil, France; 2 INSERM U955, Institut Mondor de Recherche Biomédicale, Créteil, France; 3 Equipe Opérationnelle d’Hygiène, Département de Prévention, Diagnostic et Traitement des Infections, Hôpital Henri Mondor, APHP, Créteil, France; 4 Plateforme de Génomique, Hôpital Henri Mondor 378967 , Créteil, France; 5 Ecology and Evolution of Antibiotic Resistance Unit, Institut Pasteur, CNRS UMR6047, Université Paris Cité 27058, , Paris, France; Universita degli Studi di Roma la Sapienza, Rome, Italy

**Keywords:** plasmid outbreak, gene amplification, gene dosage effect

## Abstract

Piperacillin-tazobactam (TZP) resistance in *Klebsiella pneumoniae* involves diverse mechanisms with unclear prevalence and phenotypic impact. To elucidate these mechanisms, we analyzed *K. pneumoniae* clinical isolates resistant to TZP but susceptible to cefotaxime and cefepime. Among 53 isolates, 14 were further studied by MIC testing for TZP, amoxicillin-clavulanic acid (AMC), and ceftazidime (CAZ). Short-read sequencing was performed for all 14 isolates and long-read sequencing for two. Core-genome MLST showed that all were unrelated. Two had a *bla*_OXA-1_ gene, one also carrying an *ompK35* porin gene mutation; two others had the same mutation in the promoter of the chromosomal copy of *bla*_SHV_ usually associated with overexpression. In the remaining 10, resistance correlated with plasmid-borne *bla*_SHV-1_ copies. Nine isolates carried *bla*_SHV-1v1_ in the same IS*26* pseudocompound transposon (PTn), corresponding to PTnSHV-L and located on a conserved IncFIB(K)_1_Kpn3 plasmid in eight. The tenth isolate carried PTnSHV-L with a distinct *bla*_SHV-1_ variant on both an IncHI1B_1_pNDM-MAR plasmid and a high-copy-number Col-type plasmid. Read depth analysis confirmed that *bla*_SHV_ copy number correlated with TZP, AMC, and CAZ MICs. Large-scale database screening identified related IncFIB(K)_1_Kpn3 plasmids, strongly associated with *K. pneumoniae* and frequently carrying a PTnSHV-L marker. Analysis of a *K. pneumoniae* genome data set confirmed the frequent co-occurrence of this plasmid and the PTnSHV-L marker in strains with multiple *bla*_SHV_ copies. These findings suggest the emergence of an epidemic plasmid adapted to *K. pneumoniae* and driving TZP resistance through *bla*_SHV-1_ amplification, underscoring the need for genomic surveillance to detect amplification-based resistance overlooked by standard phenotypic or PCR assays.

## INTRODUCTION

*Klebsiella pneumoniae* is a major clinical pathogen, particularly in healthcare settings. Beyond its intrinsic low-level production of the Ambler class A β-lactamase SHV, various determinants contribute to antibiotic resistance. Among them, some confer resistance to piperacillin-tazobactam (TZP), a widely used β-lactam/β-lactamase inhibitor combination. Several mechanisms are involved, including (i) non-SHV β-lactamases, such as narrow-spectrum OXA enzymes (e.g., OXA-1, OXA-10, OXA-30) (1); (ii) inhibitor-resistant SHV variants (2); (iii) mutations in the *bla*_SHV_ promoter leading to gene overexpression, sometimes associated with decreased permeability (3, 4); and (iv) acquisition of a plasmid-borne copy of *bla*_SHV_ with IS*26*-mediated tandem amplification leading to a gene dosage effect (5). Additionally, mutations at some positions (e.g., Ambler positions 179 and 238) can extend the hydrolysis spectrum of SHV β-lactamase, conferring resistance to third- and fourth-generation cephalosporins (6).

While these mechanisms are well described, their respective frequencies in clinical isolates resistant to piperacillin-tazobactam remain unclear. Furthermore, the mobile elements involved and the potential plasmid vehicles, which can represent a major threat for hospital outbreaks, have not been extensively studied. Our study aims to elucidate the molecular bases of TZP resistance through whole-genome analysis of clinical isolates at our University Hospital in Créteil, France. We also characterized in detail plasmid vehicles and, through large-scale genome database screening, explored the dissemination of a likely epidemic plasmid driving resistance dissemination.

## MATERIALS AND METHODS

### Bacterial strains and susceptibility testing

*K. pneumoniae* isolates that had been routinely collected at Henri Mondor University Hospital, Créteil, France, between 1 October 2022 and 31 December 2023, and resistant to TZP but susceptible to cefepime (FEP) and cefotaxime (CTX) by agar disk diffusion (Table S1) were examined here. Among them, randomly selected isolates were re-cultured to determine the MICs of AMC, CAZ (E-tests, Biomerieux), and TZP (MIC Test Strip, Liofilchem) by gradient diffusion strips. Antimicrobial susceptibility testing was performed and interpreted according to the CA-SFM-EUCAST 2022 recommendations.

### Whole genome sequencing and typing

Genomic DNA was extracted on a Chemagic Prime 360 robot (Revvity chemagen) and sequenced on an Illumina NovaSeq 6000 platform. Genomes were assembled using Shovill 1.0.4 (7). Contamination and completeness were assessed using CheckM v1.2.2, and only genomes with <5% of contamination and >95% of completeness were further considered (8). Genome typing was performed using Kleborate v2.1.0 (9) and Abricate (10) with the PlasmidFinder database (80% identity/coverage) (11). An all-vs-all genome comparison was conducted using core-genome MLST (cgMLST) with chewBBACA v3.3.2 (12) and the *K. pneumoniae/variicola/quasipneumoniae* cgMLST scheme (https://www.cgmlst.org/ncs/schema/Kpneumoniae_complex/), consisting of 2,358 loci.

Two isolates were also sequenced by long-read methods. After overnight growth in LB with 2 mg/L CAZ, DNA was extracted using the Monarch HMW DNA Extraction Kit for Cells & Blood (New England Biolabs) and sequenced on Oxford Nanopore Technologies (v14 chemistry) (https://plasmidsaurus.com/genome). Genomes were assembled using Trycycler (13), with Illumina reads for polishing as described by Wick et al. (14). A manual curation of these hybrid assemblies was performed by aligning Illumina reads to the assembly with breseq v0.39.0 (15) to identify positions with alternative bases within the *bla*_SHV_ regions. Genome annotation was done with Bakta v1.9.4 (16), and maps of plasmids carrying *bla*_SHV-1_ were generated using Clinker (17) and Proksee (18).

### *bla*_SHV_ analysis

To search for the presence of two different *bla*_SHV_ alleles in a given genome and determine their sequence, Illumina reads were mapped onto the *bla*_SHV-1v1_ gene (Genbank accession number AF148850.1) using breseq v0.39.0 (15), searching for positions with multiple alternative bases. For such genomes, the corresponding alleles were extracted from the BAM files using samtools and bcftools (19). The same approach was applied to the *bla*_SHV_ promoter by aligning Illumina reads to the promoter region of the RefSeq reference genome of *K. pneumoniae* HS11286 (accession number CP003200.1). Detected variants were assigned to either the chromosome or plasmid by comparing the variant-specific depth with the predicted chromosomal and plasmid depth (see below). *bla*_SHV_ nucleotide and SHV protein variants were identified using BLDB (20). For *bla*_SHV-1_ and *bla*_SHV-11_ alleles not reported in the study by Ford et al. (21), new allele identifiers were assigned by continuing the numbering scheme established in that study, beginning with *bla*_SHV-1v6_ and *bla*_SHV-11v4_.

### Sequencing depth and *bla*_SHV_ copy number

We estimated the sequencing depth of several determinants from Illumina short reads. Chromosome sequencing depth was estimated from the average read depth of the seven *K. pneumoniae* MLST genes (22). The read depths for plasmids carrying *bla*_SHV_ were estimated using the relevant PlasmidFinder replicon target, and *bla*_SHV_ sequencing depth was also determined. For pKP08-HMN-2 and pKP08-HMN-3, sequencing depth was estimated by mapping reads to a shared region of the plasmids (the first 10,000 bp of pKP08-HMN-2). The number of plasmid-borne *bla*_SHV_ copies was estimated as follows: copy number = [(mean *bla*_SHV_ depth) – (mean MLST gene depth)]/(mean MLST gene depth). Subtracting the chromosomal depth, estimated from MLST gene depth, accounts for the native chromosomal copy of *bla*_SHV_ present in *K. pneumoniae*. The number of *bla*_SHV_ copies per plasmid was computed as follows: copy number/plasmid = [(mean *bla*_SHV_ depth) – (mean MLST gene depth)]/(mean plasmid marker depth).

### Identification of closely related plasmids among complete and short-read based assemblies

To identify the plasmid of interest among the *K. pneumoniae* isolates sequenced in this study and in public databases, we applied a three-step approach. First, we used Abricate with the PlasmidFinder database to detect replicons, applying thresholds of ≥80% identity and coverage.

Next, we constructed a custom database containing all gene sequences from pKP13-HMN-1, after removing highly redundant sequences using CD-HIT v4.8.1 clustering (99% identity and coverage) (23). This database was then used to determine the proportion of matching genes present in each sequence (≥95% identity and coverage). The minimal proportion required to consider a plasmid as related was adjusted according to the type of sequence: at least 90% for complete plasmid sequences and at least 75% for short-read-based assemblies, to account for assembly fragmentation.

Finally, we performed a BLASTn search to detect a short sequence overlapping the last gene and the IS*26* in PTnSHV-L (coordinates in pKP13-HMN-1: NZ_OZ259398.1:83,057-83,208). The presence of this element was used as a proxy marker for the presence of PTnSHV-L.

Three public data sets were screened using this approach. The first, PLSDB v.2024_05_31_v2 (24), compiles all complete plasmid sequences available in the NCBI Nucleotide database as of 31 May 2024. The second, AllTheBacteria (25), comprises 2,440,377 Illumina-based bacterial whole-genome sequences available in the ENA as of August 2024. Bacterial species were obtained from the AllTheBacteria metadata. The third data set consists of *K. pneumoniae* genomes for which the number of *bla*_SHV_ copies had been predicted (26), with short reads retrieved from the SRA using fastq-dump v3.2.1 and assembled with Shovill, as described above.

## RESULTS

### TZP-resistant and CTX/FEP-susceptible strains are frequent in clinical isolates and belong to different sequence types

Over the 14-month study period, 476 *K. pneumoniae* strains were isolated from clinical samples and tested by agar disk-diffusion, among which 53 (11.1%) were resistant to TZP but susceptible to CTX and FEP. They were isolated from various samples including urine (*n* = 15), blood cultures (*n* = 12), respiratory specimens (*n* = 11), pus (*n* = 4), surgical specimens (*n* = 3), wound (*n* = 3), bile (*n* = 2), catheter (*n* = 1), drainage fluid (*n* = 1), and peritoneal fluid (*n* = 1). Fourteen of these strains (blood cultures: *n* = 10, urine: *n* = 2, wound: *n* = 1, catheter: *n* = 1), spread over the period, were randomly selected for further analysis and named KP01 to KP14 (Table S1). According to MIC test strips, seven strains were resistant to AMC, six were resistant to TZP, three fell within the area of technical uncertainty, and five were categorized as “Susceptible, increased exposure” to CAZ (Table 1).

**TABLE 1:** Genotypic and phenotypic characteristics of the strains

Strains	MLST	Non-*bla*_SHV_ genes	Porin mutation	Chromosomal *bla*_SHV_		Plasmid-borne *bla*_SHV_		*bla*_SHV_ plasmid support	Number of *bla*_SHV_ plasmid-borne copies	Number of *bla*_SHV_ copies /plasmid	Amoxicillin clavulanic acid MIC (mg/L) (SIR^*g*^)	Piperacillin tazobactam MIC (mg/L) (SIR^*g*^)	Ceftazidime MIC (mg/L) (SIR^*g*^)
				−10 box (reference = ACAAAT)	Allele	Promoter mutation (−10 box)	Allele						
KP06	307	*bla*_OXA-1_	OmpK35 (Y117X)	ACAAAT	*bla*_SHV-28-like_ *c*	NA	NA*^d^*	NA	NA	NA	>256 (R)	192 (R)	0.5 (S)
KP07	86	*bla*_OXA-1_	–^*h*^	ACAAAT	*bla*_SHV-1v6_	NA	NA	NA	NA	NA	12 (R)	6 (S)	0.094 (S)
KP04	2715	–	–	AAAAAT	*bla*_SHV-1v3_	NA	NA	NA	NA	NA	4 (S)	6 (S)	0.5 (S)
KP05	45-2LV^*b*^	–	–	AAAAAT	*bla*_SHV-11v4_	NA	NA	NA	NA	NA	6 (S)	16 (ATU)	0.75 (S)
KP01	70	–	–	ACAAAT	*bla*_SHV-32_	AAAAAT	*bla*_SHV-1v1_	unknown	1.5	Undetermined^*f*^	12 (R)	16 (ATU)	1 (S)
KP02	13	–	–	ACAAAT	*bla*_SHV-1v7_	AAAAAT	*bla*_SHV-1v1_	IncFIB(K)_1_Kpn3	22.6	13.8	>256 (R)	>256 (R)	3 (I)
KP03	870	–	–	ACAAAT	*bla*_SHV-1v8_	AAAAAT	*bla*_SHV-1v1_	IncFIB(K)_1_Kpn3	1.3	0.8	8 (S)	24 (R)	0.75 (S)
KP09	35	–	–	ACAAAT	*bla*_SHV-33_	AAAAAT	*bla*_SHV-1v1_	IncFIB(K)_1_Kpn3	1.8	0.8	6 (S)	4 (S)	0.38 (S)
KP10	45	–	–	ACAAAT	*bla*_SHV-1v3_	AAAAAT	*bla*_SHV-1v1_	IncFIB(K)_1_Kpn3	0.8	0.6	4 (S)	3 (S)	0.38 (S)
KP11	45	–	–	ACAAAT	*bla*_SHV-1v3_	AAAAAT	*bla*_SHV-1v1_	IncFIB(K)_1_Kpn3	49.2	30.9	>256 (R)	>256 (R)	3 (I)
KP12	35	–	–	ACAAAT	*bla*_SHV-33_	AAAAAT	*bla*_SHV-1v1_	IncFIB(K)_1_Kpn3	1.3	1.1	8 (S)	16 (ATU)	1.5 (I)
KP13^*a*^	45	–	–	ACAAAT	*bla*_SHV-1v3_	AAAAAT	*bla*_SHV-1v1_	IncFIB(K)_1_Kpn3	8.1	5.2/5^*e*^	128 (R)	>256 (R)	4 (I)
KP14	45	–	–	ACAAAT	*bla*_SHV-1v3_	AAAAAT	*bla*_SHV-1v1_	IncFIB(K)_1_Kpn3	1.2	0.8	6 (S)	4 (S)	0.38 (S)
KP08^*a*^	567	–	–	ACAAAT	*bla*_SHV-11v4_	AAAAAT	*bla*_SHV-1v9_	IncHI1B_1_pNDM-MAR	29	2^*e*^	48 (R)	>256 (R)	2 (I)
						AAAAAT	*bla*_SHV-1v9_	Col440II_1/ColRNAI_1		1^*e*^			

^*a*^ These isolates were long-read sequenced.

^*b*^ 2LV, two locus variants.

^*c*^ No perfect nucleotide match was found, but the translated protein differs from the SHV-28 allele by a single amino acid substitution (G232E).

^*d*^ NA, not applicable.

^*e*^ Number of copies according to long-read sequencing.

^*f*^ The copy number per plasmid could not be determined because the plasmid support remains unknown.

^*g*^ S, susceptible; I, susceptible, increased exposure; R, resistant; ATU, area of technical uncertainty.

^*h*^ –, no gene or mutation detected.

### Studied strains carry a distinct plasmid-borne copy of *bla*_SHV-1_ and are genetically unrelated

Ten different sequence types (STs) were identified from short-read sequencing. cgMLST showed that all strains differed by more than 10 alleles (median = 1,791.0, IQR = 51), even those belonging to the same ST (ST35 [*n*=2] and ST45 [*n*=4]) (Fig. S1), ruling out a clonal outbreak.

From whole genome sequence data, KP06 and KP07 had *bla*_OXA-1_ (Table 1). KP06 also had a mutation in the major porin gene *ompK35* (C351A), leading to a truncated OmpK35 protein (Y117X), which may explain its higher level of resistance to TZP (Table 1). KP04 had a 1 bp deletion between the ribosome binding site and the start codon of *bla*_SHV_ and both KP04 and KP05 had the same mutation in the −10 box of the *bla*_SHV_ promoter region (ACAAAT>AAAAAT) (Table 1; Fig. S2). This mutation has been shown to increase *bla*_SHV_ expression and is occasionally observed in chromosomal copies and consistently present in plasmid-borne ones (3, 4).

Analysis and comparison of Illumina short reads sequencing depth across the 14 isolates revealed increased *bla*_SHV_ sequencing depth relative to the chromosome in the remaining 10 isolates (Table 1; Fig. S3). Each carried two *bla*_SHV_ variants, suggesting a plasmid-borne copy. Nine of 10 had *bla*_SHV-1v1_, a *bla*_SHV-1_ allele commonly found on plasmids (21), and the tenth had a novel *bla*_SHV-1_ allele (Table 1; Data S1). *bla*_SHV_ chromosomal copies were *bla*_SHV-1_ (*n*=6), *bla*_SHV-33_ (*n*=2), *bla*_SHV-11_ (*n*=1) and *bla*_SHV-32_ (*n*=1). Interestingly, strains with the highest MICs for AMC, TZP, and CAZ (KP02, KP08, KP11, KP13) carried the highest *bla*_SHV_ copy numbers, ranging from 8.1 to 49.2 copies (Table 1; Fig. S4).

### Plasmid-borne copies of *bla*_SHV-1_ are often associated with the same plasmid backbone

**Fig 1:**
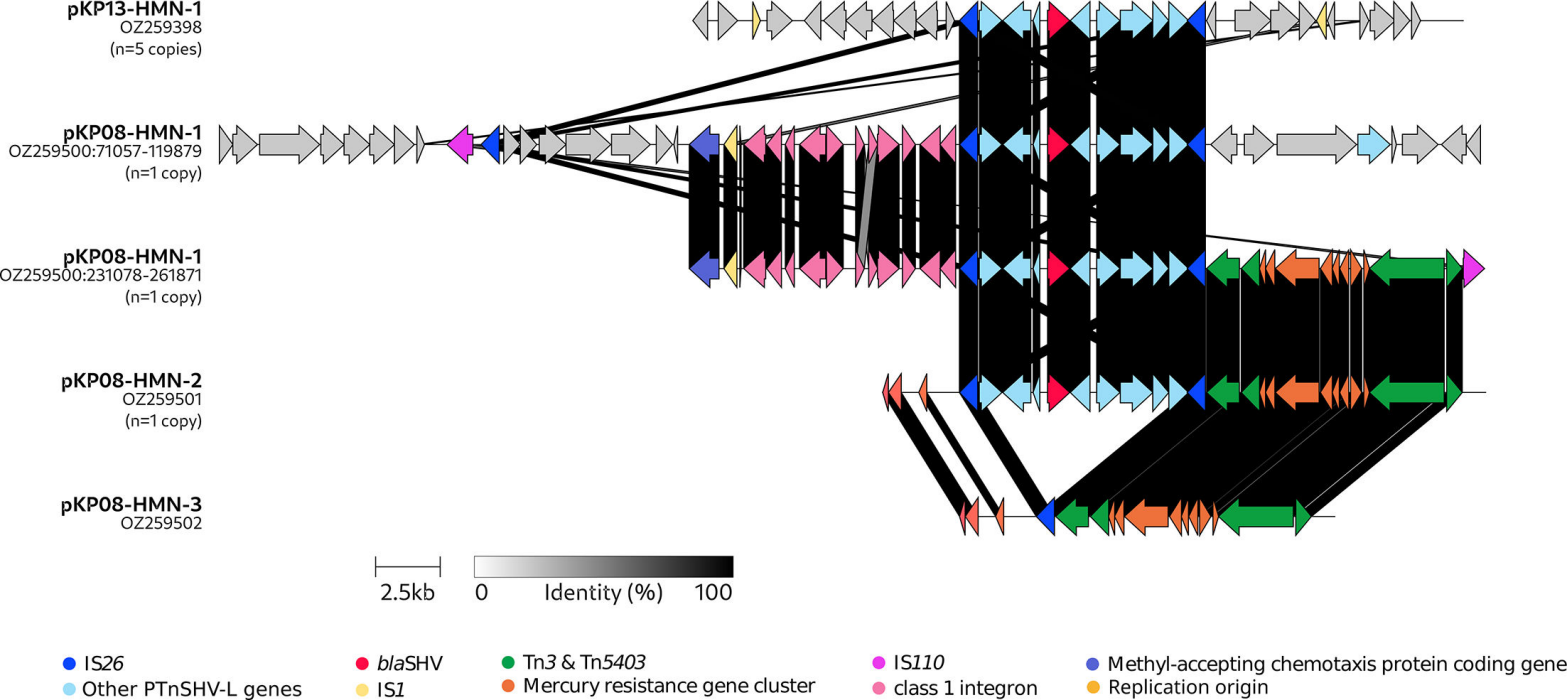
Alignment of plasmid regions containing PTnSHV-L. Colored arrows show different types of genes, as indicated in the key. Shading between diagrams indicates the level of protein identity (minimum 60%). The number of PTnSHV-L copies in each plasmid is shown under its name on the left. For complete maps of pKP08-HMN-1, pKP08-HMN-2, and pKP13-HMN-1, see Fig. S5.

Long-read sequencing of KP08 and KP13, which had the highest TZP and CAZ MICs, different *bla*_SHV-1_ alleles and replicon types (Table 1), revealed distinct *bla*_SHV_ on distinct plasmids. KP13 had a 207,526 bp IncFIB(K)_1_Kpn3 plasmid (pKP13-HMN-1) and a 5,542 bp plasmid (pKP13-HMN-2). pKP13-HMN-1 carried five identical copies of an IS*26* flanked PTn (Fig. 1; Fig. S5A), corresponding to PTnSHV-L (9,636-pb, accession number CP000603) (27) with *bla*_SHV-1v1._ The presence of these five copies is consistent with IS*26*-mediated amplification.

KP08 had five plasmids: a 266,042 bp IncHI1B_1_pNDM-MAR plasmid (pKP08-HMN-1), two ColRNA_1/Col440II_1 of 23,364 bp (pKP08-HMN-2) and 14,547 bp (pKP08-HMN-3), a 5,229 bp Col440I_1 plasmid (pKP08-HMN-4), and a 4,389 bp Col440II_1 (pKP08-HMN-5) (Fig. 1; Fig. S5B). On pKP08-HMN-1, two PTnSHV-L were identified, each adjacent to another IS*26* PTn containing a class one integron, thus forming two PTn arrays. PTnSHV-L was also detected on pKP08-HMN-2. Of note, pKP08-HMN-3 was identical to pKP08-HMN-2, except that pKP08-HMN-3 had a single copy of IS*26* instead of PTnSHV-L at the same position. We predicted 29 plasmid-borne copies of *bla*_SHV-1_ based on read-depth analysis. However, according to long-read sequencing, this high copy number did not result from an IS*26*-mediated amplification, but from the high copy number of pKP08-HMN-2. Indeed, the ColRNA_1/Col440II_1 plasmids (pKP08-HMN-2 and pKP08-HMN-3) showed a relative depth of 29×. Hence, considering a chromosomal copy, two copies on pKP08-HMN-1, and a single copy on pKP08-HMN-2, we estimated that pKP08-HMN-2 represented 91% of the ColRNA_1/Col440II_1 plasmids.

**Fig 2:**
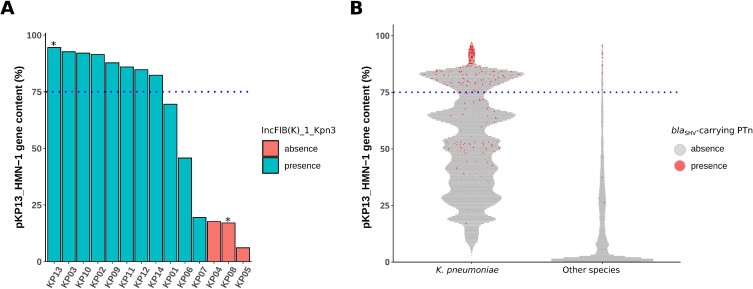
Screening for pKP13-HMN-1-related plasmids among short-read–based genome assemblies. (**A**) Percentage of genes from pKP13-HMN-1 (the plasmid from isolate KP13) identified in the 14 *K. pneumoniae* isolates analyzed in this study. Bar plots are colored according to the presence or absence of the IncFIB(K)_1_Kpn3 marker. Asterisks indicate isolates sequenced with long-read technology. (**B**) Percentage of pKP13-HMN-1 genes identified in IncFIB(K)_1_Kpn3-positive genomes (*n* = 89,531) from the AllTheBacteria database. Species assignments were retrieved from the AllTheBacteria metadata. Each point represents a genome, and the presence or absence of the PTnSHV-L marker is highlighted in red or grey, respectively. In both panels, the blue dashed line indicates the 75% threshold used to define the presence of a pKP13-HMN-1-related plasmid in short-read-based assemblies.

As nine isolates carried the same *bla*_SHV-1v1_ allele and the IncFIB(K)_1_Kpn3 plasmid marker found in KP13, we searched for pKP13-HMN-1-related plasmids among short-read assemblies. We found more than 75% of pKP13-HMN-1 genes in 8 out of 14 isolates, all carrying *bla*_SHV-1v1_ and matching a PTnSHV-L marker sequence (Fig. 2A). Of note, although it carried the same plasmid-borne *bla*_SHV-1v1_ allele, isolate KP01 harbored fewer pKP13-HMN-1 genes, which may reflect a more distantly related IncFIB(K)_1_Kpn3 plasmid. We then estimated the number of *bla*_SHV_ copies per plasmid from Illumina short reads and identified three strains with more than five copies: KP02, KP11, and KP13 (Table 1).

### Plasmids similar to pKP13-HMN-1 are identified in public databases and are specifically associated with *K. pneumoniae*

Next, we searched for plasmids similar to pKP13-HMN-1 in PLSDB, a large public database of complete plasmid sequences. Among the 72,556 available plasmids, 1,927 carried the replicon marker IncFIB(K)_1_Kpn3. Screening for gene content identified 10 highly similar plasmids that carried more than 90% of the pKP13-HMN-1 genes (Table S2). Of them, one had two PTnSHV-L with *bla*_SHV-1v1_, seven had one copy, and two had none. Plasmid alignment confirmed their closeness (Fig. S6).

Then, we analyzed public data sets to assess the prevalence of pKP13-HMN-1-like plasmids in large genomic databases. First, we screened the AllTheBacteria database, which includes over 2,400,000 short-read-based bacterial genome assemblies. We found an IncFIB(K)_1_Kpn3 replicon in 89,531 genomes (3.67%), among which 53,004 (59.20%) were assigned to *K. pneumoniae* and 36,527 (40.80%) to other species. Among the *K. pneumoniae* genomes, 12,165 (22.95%) contained at least 75% of the pKP13-HMN-1 gene content, whereas only 511 (1.40%) of non-*K*. *pneumoniae* genomes carried a related plasmid (Fig. 2B; Table S3). The concomitant presence of the PTnSHV-L marker was found in 941/12,165 (7.74%) of *K. pneumoniae* genomes (Table S3) and 43/511 (8.41%) of non-*K*. *pneumoniae* genomes, including 8/43 (18.60%) belonging to other *Klebsiella* species (Fig. 2B; Table S4). Of note, among *K. pneumoniae* genomes carrying both the pKP13-HMN-1-related plasmid and PTnSHV-L, 19.91**%** belonged to ST45.

Finally, we screened a second data set composed exclusively of *K. pneumoniae* genomes, in which the number of *bla*_SHV_ copies had been previously predicted (26). Among the 3,960 genomes retrieved, the PTnSHV-L marker was detected in 86/416 genomes with more than one *bla*_SHV_ copy, compared to 8/3,544 genomes with a single copy (Fisher’s Exact Test *P* < 0.001). Overall, 85/3,960 (2.15%) genomes carried both a pKP13-HMN-1-related plasmid and the PTnSHV-L marker. Among them, 78 (91.76%), 49 (57.65%), and 25 (29.41%) harbored more than one, two, and five *bla*_SHV_ copies, respectively. Genomes with multiple *bla*_SHV_ copies and carrying both determinants represented 18.75% of all *bla*_SHV_ multicopy genomes, with 29 (37.18%) belonging to ST45.

In sum, the concomitant presence of an IncFIB(K)_1_Kpn3 plasmid similar to pKP13-HMN-1 and PTnSHV-L was observed across large genomic data sets, almost exclusively among *K. pneumoniae* isolates and frequently associated with ST45. This association was often linked to an increased copy number of *bla*_SHV_.

## DISCUSSION

Using a collection of *K. pneumoniae* isolates from routine clinical samples, we sought to characterize TZP resistance. While several mechanisms were observed, the main driver was plasmid-borne copies of *bla*_SHV-1_, identified in 10/14 of our strains. As recently reported by others (26), the *bla*_SHV_ copy number correlates with the level of antibiotic resistance. Nevertheless, CAZ MICs remained low even in isolates with high copy numbers, consistent with the absence of ESBL-associated mutations in our strains. However, as suggested by Hammond et al. (28), the occurrence of multiple plasmid-borne copies may be clinically significant, as it could represent an intermediate step toward the subsequent selection of ESBL-associated mutations, which in combination with high copy number lead to high-level resistance to 3rd and 4th-generation cephalosporins.

In our study, unrelated strains had the same *bla*_SHV-1v1_ allele and IncFIB(K)_1_Kpn3 marker. The *bla*_SHV-1v1_ genes were located in PTnSHV-L, an IS*26*-containing element, which enables the formation of a translocatable unit capable of targeted conservative reactions leading to a PTn amplification and, consequently, increased copy number (27). Screening of large-scale plasmid and genome databases confirmed the presence of similar plasmids, particularly associated with *K. pneumoniae* and frequently carrying a marker of PTnSHV-L. As observed in our clinical isolates, several different *K. pneumoniae* STs harbored such plasmids, among which ST45 appeared as one of the most frequent. Moreover, using a previously published data set (26), we confirmed the association of the PTnSHV-L marker and the presence of multiple *bla*_SHV_ copies, as well as the relatively high proportion of pKP13-HMN-1-like plasmids among strains carrying *bla*_SHV_ multiple copies. Together, these data strongly support the existence of an epidemic plasmid spreading within the *K. pneumoniae* population.

Interestingly, plasmids with an IncFIB(K)_1_Kpn3 replicon have been described in other studies and are known to be highly associated with *K. pneumoniae*, consistent with the high prevalence in *K. pneumoniae* genomes from AllTheBacteria database (72.65%). As an example, one of these plasmids, pKPN-307, carries the ESBL gene *bla*_CTX-M-15_ and several virulence-associated genes and has been proposed as a key player in the adaptation, evolution, and dissemination of the globally distributed ST307 high-risk clone (29, 30). Hence, these plasmids are a versatile genetic support that may enable efficient diffusion of resistance determinants within *K. pneumoniae*.

We also report a previously undescribed location of *bla*_SHV_. One of our isolates carried *bla*_SHV_ both on an IncHI1 plasmid and a ColRNA_1/Col440II_1 plasmid. This finding highlights the high mobility mediated by IS*26* and confirms the importance of two pathways contributing to increased resistance through a multicopy strategy: (i) IS*26-*mediated amplification of β-lactamase genes and (ii) transposition to high-copy-number plasmids (5). The identical *bla*_SHV-1_ allele observed in both plasmids, and the presence of an IS*26* on the colRNA_1/col440II_1 that lacked *bla*_SHV-1_ (pKP08-HMN-3), supports the hypothesis of gene mobilization from one plasmid to the other via an IS*26*-mediated targeted conservative reaction, rather than acquisition of two different plasmids each carrying *bla*_SHV-1_. Further work is needed to characterize and determine the particularity and the frequency of such events.

Our study has several limitations, including the limited number of isolates, all originating from a single hospital, and their random selection. Broader epidemiological studies are needed to confirm the prevalence of these resistance mechanisms and limit bias. To mitigate these limitations, we conducted a large-scale database screening. Another limitation lies in strain selection, which was based on disk diffusion testing. Indeed, when tested using MIC test strips, many isolates exhibited only low-level or even borderline susceptibility to TZP. Further studies are needed to determine the most accurate method for detecting strains harboring plasmid-borne copies of *bla*_SHV_ (e.g., disk diffusion, MIC test strips, or broth microdilution), particularly those with a low *bla*_SHV_ copy number that may increase under antibiotic treatment. Indeed, while we did not conduct additional phenotypic analyses to evaluate such variations under antibiotic pressure, our data show a clear correlation between higher copy number and increased MICs, and similar adaptive amplification mechanisms have been demonstrated for other SHV β-lactamases (31, 32) as well as other enzymes (5, 33). Moreover, from a technical point of view, our results also echo the known difficulty of accurately determining TZP susceptibility, which strongly depends on the testing method, as shown in the MERINO trial and its post hoc analysis (34, 35). Estimating copy number from short reads may lead to inaccuracies. However, we observed very similar values for the two strains sequenced both using short-read and long-read technologies, suggesting a limited margin of error. Finally, detecting a plasmid similar to pKP13_HMN-1 from short-read assemblies is challenging due to the fragmentation inherent to Illumina sequencing. Combining plasmid gene content analysis with the detection of the PTnSHV-L marker provides an interesting proxy for its presence across genomes. Long-read sequencing data would clearly improve the analysis, but such data are currently limited in available databases.

In conclusion, multiple mechanisms contribute to TZP resistance in *K. pneumoniae* that do not produce ESBL. Within our data set, the primary driver of resistance appeared to be plasmid-borne *bla*_SHV-1_ copies, frequently carried on an epidemic plasmid, rather than the presence of additional β-lactamases such as narrow-spectrum *bla*_OXA-1_ or mutations in *bla*_SHV_ promoter. The high mobility conferred by IS*26* may also facilitate the integration of PTnSHV-L into small, high-copy-number Col plasmids, thereby further enhancing β-lactam resistance. This underscores the need for broader surveillance to monitor the genetic diversity and mobility of *bla*_SHV_ in *K. pneumoniae*. It also highlights the role of gene amplification in resistance, an adaptive mechanism that is difficult to detect in routine clinical laboratories using both phenotypic tests and multiplex PCR targeting specific genes or variants.

## Data Availability

Short- and long-read sequences are available under BioProject PRJEB88171. Long-read assemblies for KP13 are OZ259397.1 (chromosome), OZ259398.1 (pKP13-HMN-1), and OZ259399.1 (pKP13-HMN-2). For KP08, long-read assemblies are OZ259499.2 (chromosome), OZ259500.1 (pKP08-HMN-1), OZ259501.1 (pKP08-HMN-2), OZ259502.2 (pKP08-HMN-3), OZ259503.1 (pKP08-HMN-4), and OZ259504.1 (pKP08-HMN-5).

## SUPPLEMENTAL MATERIAL

Data S1 aac.01682-25-s0001.docx Nucleotide sequences of *bla*_SHV_ alleles identified in KP01 to KP14.

Supplemental figures aac.01682-25-s0002.pdf Fig. S1 to S6.

Supplemental tables aac.01682-25-s0003.xlsx Tables S1 to S4.

ASM does not own the copyrights to Supplemental Material that may be linked to, or accessed through, an article. The authors have granted ASM a non-exclusive, world-wide license to publish the Supplemental Material files. Please contact the corresponding author directly for reuse.
