# Supplementary material for: Piperacillin-tazobactam resistance in Klebsiella pneumoniae is often associated with IS26-mediated blaSHV-1 amplification in a widespread Klebsiella-adapted plasmid
Source: Antimicrob Agents Chemother. 2026 Mar 24;70(5):e01682-25. doi: 10.1128/aac.01682-25 (PMC13148056; doi:10.1128/aac.01682-25)
Supplement: Data S1 — Nucleotide sequences of blaSHV alleles identified in KP01 to KP14. [file aac.01682-25-s0001.docx]

Supplementary file 1. Nucleotide sequences of blaSHV alleles identified in KP01 to KP14.

>blaSHV-1v1

ATGCGTTATATTCGCCTGTGTATTATCTCCCTGTTAGCCACCCTGCCGCTGGCGGTACACGCCAGCCCGCAGCCGCTTGAGCAAATTAAACTAAGCGAAAGCCAGCTGTCGGGCCGCGTAGGCATGATAGAAATGGATCTGGCCAGCGGCCGCACGCTGACCGCCTGGCGCGCCGATGAACGCTTTCCCATGATGAGCACCTTTAAAGTAGTGCTCTGCGGCGCAGTGCTGGCGCGGGTGGATGCCGGTGACGAACAGCTGGAGCGAAAGATCCACTATCGCCAGCAGGATCTGGTGGACTACTCGCCGGTCAGCGAAAAACACCTTGCCGACGGCATGACGGTCGGCGAACTCTGCGCCGCCGCCATTACCATGAGCGATAACAGCGCCGCCAATCTGCTACTGGCCACCGTCGGCGGCCCCGCAGGATTGACTGCCTTTTTGCGCCAGATCGGCGACAACGTCACCCGCCTTGACCGCTGGGAAACGGAACTGAATGAGGCGCTTCCCGGCGACGCCCGCGACACCACTACCCCGGCCAGCATGGCCGCGACCCTGCGCAAGCTGCTGACCAGCCAGCGTCTGAGCGCCCGTTCGCAACGGCAGCTGCTGCAGTGGATGGTGGACGATCGGGTCGCCGGACCGTTGATCCGCTCCGTGCTGCCGGCGGGCTGGTTTATCGCCGATAAGACCGGAGCTGGCGAGCGGGGTGCGCGCGGGATTGTCGCCCTGCTTGGCCCGAATAACAAAGCAGAGCGCATTGTGGTGATTTATCTGCGGGATACCCCGGCGAGCATGGCCGAGCGAAATCAGCAAATCGCCGGGATCGGCGCGGCGCTGATCGAGCACTGGCAACGCTAA

>blaSHV-1v3

ATGCGTTATATTCGCCTGTGTATTATCTCCCTGTTAGCCACCCTGCCGCTGGCGGTACACGCCAGCCCGCAGCCGCTTGAGCAAATTAAACTAAGCGAAAGCCAGCTGTCGGGCCGCGTAGGCATGATAGAAATGGATCTGGCCAGCGGCCGCACGCTGACCGCCTGGCGCGCCGATGAACGCTTTCCCATGATGAGCACCTTTAAAGTAGTGCTCTGCGGCGCAGTGCTGGCGCGGGTGGATGCCGGTGACGAACAGCTGGAGCGAAAGATCCACTATCGCCAGCAGGATCTGGTGGACTACTCGCCGGTCAGCGAAAAACACCTTGCCGACGGCATGACGGTCGGCGAACTCTGCGCCGCCGCCATTACCATGAGCGATAACAGCGCCGCCAATCTGCTGCTGGCCACCGTCGGCGGCCCCGCAGGATTGACTGCCTTTTTGCGCCAGATCGGCGACAACGTCACCCGCCTTGACCGCTGGGAAACGGAACTGAATGAGGCGCTTCCCGGCGACGCCCGCGACACCACTACCCCGGCCAGCATGGCCGCGACCCTGCGCAAGCTGCTGACCAGCCAGCGTCTGAGCGCCCGTTCGCAACGGCAGCTGCTGCAGTGGATGGTGGACGATCGGGTCGCCGGACCGTTGATCCGCTCCGTGCTGCCGGCGGGCTGGTTTATCGCCGATAAGACCGGAGCTGGCGAGCGGGGTGCGCGCGGCATTGTCGCCCTGCTTGGCCCGAATAACAAAGCAGAGCGCATTGTGGTGATTTATCTGCGGGATACGCCGGCGAGCATGGCCGAGCGAAATCAGCAAATCGCCGGGATCGGCGCGGCGCTGATCGAGCACTGGCAACGCTAA

>blaSHV-32

ATGCGTTATATTCGCCTGTGTATTATCTCCCTGTTAGCCACCCTGCCGCTGGCGGTACACGCCAGCCCGCAGCCGCTTGAGCAAATTAAACTAAGCGAAAGCCAGCTGTCGGGCCGCGTAGGCATGATAGAAATGGATCTGGCCAGCGGCCGCACGCTGACCGCCTGGCGCGCCGATGAACGCTTTCCCATGATGAGCACCTTTAAAGTAGTGCTCTGCGGCGCAGTGCTGGCGCGGGTGGATGCCGGTGACGAACAGCTGGAGCGAAAGATCCACTATCGCCAGCAGGATCTGGTGGACTACTCGCCGGTCAGCGAAAAACATCTTGCCGACGGCATGACGGTCGGCGAACTCTGTGCCGCCGTCATTACCATGAGCGATAACAGCGCCGCCAATCTGCTGCTGGCCACCGTCGGCGGCCCCGCAGGATTGACTGCCTTTTTGCGCCAGATCGACGACAACGTCACCCGCCTTGACCGCTGGGAAACGGAACTGAATGAGGCGCTTCCCGGCGACGCCCGCGACACCACTACCCCGGCCAGCATGGCCGCGACCCTGCGCAAGCTGCTGACCAGCCAGCGTCTGAGCGCCCGTTCGCAACGGCAGCTGCTGCAGTGGATGGTGGACGATCGGGTCGCCGGACCGTTGATCCGCTCCGTGCTGCCGGCGGGCTGGTTTATCGCCGATAAGACCGGAGCTGGCGAGCGGGGTGCGCGCGGGATTGTCGCCCTGCTTGGCCCGAATAACAAAGCAGAGCGCATTGTGGTGATTTATCTGCGGGATACGCCGGCGAGCATGGCCGAGCGAAATCAGCAAATCGCCGGGATCGGCGCGGCGCTGATCGAGCACTGGCAACGCTAA

>blaSHV-1v7

ATGCGTTATATTCGCCTGTGTATTATCTCCCTGTTAGCCACCCTGCCGCTGGCGGTACACGCCAGCCCGCAGCCGCTTGAGCAAATTAAACTAAGCGAAAGCCAGCTGTCGGGCCGCGTAGGCATGATAGAAATGGATCTGGCCAGCGGCCGCACGCTGACCGCCTGGCGCGCCGATGAACGCTTTCCCATGATGAGCACCTTTAAAGTAGTGCTCTGCGGCGCAGTGCTGGCGCGGGTGGATGCCGGTGACGAACAGCTGGAGCGAAAGATCCACTATCGCCAGCAGGATCTGGTGGACTACTCGCCGGTCAGCGAAAAACACCTTGCCGACGGCATGACGGTCGGCGAACTCTGCGCCGCCGCCATTACCATGAGCGATAACAGCGCCGCCAATCTGCTGCTGGCCACCGTCGGCGGCCCCGCAGGATTGACTGCCTTTTTGCGCCAGATCGGCGACAACGTCACCCGCCTTGACCGCTGGGAAACGGAACTGAATGAGGCGCTTCCCGGCGACGCCCGCGACACCACTACCCCGGCCAGCATGGCCGCGACCCTGCGCAAGCTGCTGACCAGCCAGCGTCTGAGCGCCCGTTCGCAACGGCAGCTGCTGCAGTGGATGGTGGACGATCGGGTCGCCGGACCGTTGATCCGCTCCGTGCTGCCGGCGGGCTGGTTTATCGCCGATAAGACCGGAGCTGGCGAACGGGGTGCGCGCGGCATTGTCGCCCTGCTTGGCCCGAATAACAAAGCAGAGCGCATTGTGGTGATATATCTGCGGGATACGCCGGCGAGCATGGCCGAGCGAAATCAGCAAATCGCCGGGATCGGCGCGGCGCTGATCGAGCACTGGCAACGCTAA

>blaSHV-1v8

ATGCGTTATATTCGCCTGTGTATTATCTCCCTGTTAGCCACCCTGCCGCTGGCGGTACACGCCAGCCCGCAGCCGCTTGAGCAAATTAAACTAAGCGAAAGCCAGCTGTCGGGCCGCGTAGGCATGATAGAAATGGATCTGGCCAGCGGCCGCACGCTGACCGCCTGGCGCGCCGATGAACGCTTTCCCATGATGAGCACCTTTAAAGTAGTGCTCTGCGGCGCAGTGCTGGCGCGGGTGGATGCCGGTGACGAACAGCTGGAGCGAAAGATCCACTATCGCCAGCAGGATCTGGTGGACTACTCGCCGGTCAGCGAAAAACATCTTGCCGACGGCATGACGGTCGGCGAACTCTGCGCCGCCGCCATTACCATGAGCGATAACAGCGCCGCCAATCTGCTGCTGGCCACCGTCGGCGGCCCCGCAGGATTGACTGCCTTTTTGCGCCAGATCGGCGACAACGTCACCCGCCTTGACCGCTGGGAAACGGAACTGAATGAGGCGCTTCCCGGCGACGCCCGCGACACCACTACCCCGGCCAGCATGGCCGCGACCCTGCGCAAGCTGCTGACCAGCCAGCGTCTGAGCGCCCGTTCGCAACGGCAGCTGCTGCAGTGGATGGTGGACGATCGAGTCGCCGGACCGTTGATCCGCTCCGTGCTGCCGGCGGGCTGGTTTATCGCCGATAAGACCGGAGCTGGCGAACGGGGTGCGCGCGGGATTGTCGCCCTGCTTGGCCCGAATAACAAAGCAGAGCGCATCGTGGTGATTTATCTGCGGGATACCCCGGCGAGCATGGCCGAGCGAAATCAGCAAATCGCCGGGATCGGCGCGGCGCTGATCGAGCACTGGCAACGCTAA

>blaSHV-11-v4

ATGCGTTATATTCGCCTGTGTATTATCTCCCTGTTAGCCACCCTGCCGCTGGCGGTACACGCCAGCCCGCAGCCGCTTGAGCAAATTAAACAAAGCGAAAGCCAGCTGTCGGGCCGCGTAGGCATGATAGAAATGGATCTGGCCAGCGGCCGCACGCTGACCGCCTGGCGCGCCGATGAACGCTTTCCCATGATGAGCACCTTTAAAGTAGTGCTCTGCGGCGCAGTGCTGGCGCGGGTGGATGCCGGTGACGAACAGCTGGAGCGAAAGATCCACTATCGCCAGCAGGATCTGGTGGACTACTCGCCGGTCAGCGAAAAACACCTTGCCGACGGCATGACGGTCGGCGAACTCTGTGCCGCCGCCATTACCATGAGCGATAACAGCGCCGCCAATCTGCTGCTGGCCACCGTCGGCGGCCCCGCAGGATTGACTGCCTTTTTGCGCCAGATCGGCGACAACGTCACCCGCCTTGACCGCTGGGAAACGGAACTGAATGAGGCGCTTCCCGGCGACGCCCGCGACACCACTACCCCGGCCAGCATGGCCGCGACCCTGCGCAAGCTGCTGACCAGCCAGCGTCTGAGCGCCCGTTCGCAACGGCAGCTGCTGCAGTGGATGGTGGACGATCGGGTCGCCGGACCGTTGATCCGCTCCGTGCTGCCGGCGGGCTGGTTTATCGCCGATAAGACCGGAGCTGGCGAACGGGGTGCGCGCGGGATTGTCGCCCTGCTTGGCCCGAATAACAAAGCAGAGCGCATCGTGGTGATTTATCTGCGGGATACGCCGGCGAGCATGGCCGAGCGAAATCAGCAAATCGCCGGGATCGGCGCGGCGCTGATCGAGCACTGGCAACGCTAA

>blaSHV-28-like

ATGCGTTTTATTCGCCTGTGTATTATCTCCCTGTTAGCCACCCTGCCGCTGGCGGTACACGCCAGCCCGCAGCCGCTTGAGCAAATTAAACTAAGCGAAAGCCAGCTGTCGGGCCGCGTAGGCATGATAGAAATGGATCTGGCCAGCGGCCGCACGCTGACCGCCTGGCGCGCCGATGAACGCTTTCCCATGATGAGCACCTTTAAAGTAGTGCTCTGCGGCGCAGTGCTGGCGCGGGTGGATGCCGGTGACGAACAGCTGGAGCGAAAGATCCACTATCGCCAGCAGGATCTGGTGGACTACTCGCCGGTCAGCGAAAAACATCTTGCCGACGGCATGACGGTCGGCGAACTCTGCGCCGCCGCCATTACCATGAGCGATAACAGCGCCGCCAATCTGCTGCTGGCCACCGTCGGCGGCCCCGCAGGATTGACTGCCTTTTTGCGCCAGATCGGCGACAACGTCACCCGCCTTGACCGCTGGGAAACGGAACTGAATGAGGCGCTTCCCGGCGACGCCCGCGACACCACTACCCCGGCCAGCATGGCCGCGACCCTGCGCAAGCTGCTGACCAGCCAGCGTCTGAGCGCCCGTTCGCAACGGCAGCTGCTGCAGTGGATGGTGGACGATCGAGTCGCCGGACCGTTGATCCGCTCCGTGCTGCCGGCGGGCTGGTTTATCGCCGATAAGACCGAAGCTGGCGAACGGGGTGCGCGCGGGATTGTCGCCCTGCTTGGCCCGAATAACAAAGCAGAGCGCATCGTGGTGATTTATCTGCGGGATACCCCGGCGAGCATGGCCGAGCGAAATCAGCAAATCGCCGGGATCGGCGCGGCGCTGATCGAGCACTGGCAACGCTAA

>blaSHV-1v6

ATGCGTTATATTCGCCTGTGTATTATCTCCCTGTTAGCCACCCTGCCGCTGGCGGTACACGCCAGCCCGCAGCCGCTTGAGCAAATTAAACTAAGCGAAAGCCAGCTGTCGGGCCGCGTAGGCATGATAGAAATGGATCTGGCCAGCGGCCGCACGCTGACCGCCTGGCGCGCCGATGAACGCTTTCCCATGATGAGCACCTTTAAAGTAGTGCTCTGCGGCGCAGTGCTGGCGCGGGTGGATGCCGGTGACGAACAGCTGGAGCGAAAGATCCACTATCGCCAGCAGGATCTGGTGGACTACTCGCCGGTCAGCGAAAAACATCTTGCCGACGGCATGACGGTCGGCGAACTCTGCGCCGCCGCCATTACCATGAGCGATAACAGCGCCGCCAATCTGCTGCTGGCCACCGTCGGCGGCCCCGCAGGATTGACTGCCTTTTTGCGCCAGATCGGCGACAACGTCACCCGCCTTGACCGCTGGGAAACGGAACTGAATGAGGCGCTTCCCGGCGACGCCCGCGACACCACTACCCCGGCCAGCATGGCCGCGACCCTGCGCAAGCTGCTGACCAGCCAGCGTCTGAGCGCCCGTTCGCAACGGCAGCTGCTGCAGTGGATGGTGGACGATCGAGTCGCCGGACCGTTGATCCGCTCCGTGCTGCCGGCGGGCTGGTTTATCGCCGATAAGACCGGAGCTGGCGAACGGGGTGCGCGCGGGATTGTCGCCCTGCTTGGCCCGAATAACAAAGCAGAGCGCATCGTGGTGATTTATCTGCGGGATACGCCGGCGAGCATGGCCGAGCGAAATCAGCAAATCGCCGGGATCGGCGCGGCGCTGATCGAGCACTGGCAACGCTAA

>blaSHV-1v9

ATGCGTTATATTCGCCTGTGTATTATCTCCCTGTTAGCCACCCTGCCGCTGGCGGTACACGCCAGCCCGCAGCCGCTTGAGCAAATTAAACTAAGCGAAAGCCAGCTGTCGGGCCGCGTAGGCATGATAGAAATGGATCTGGCCAGCGGCCGCACGCTGACCGCCTGGCGCGCCGATGAACGCTTTCCCATGATGAGCACCTTTAAAGTAGTGCTCTGCGGCGCAGTGCTGGCGCGGGTGGATGCCGGTGACGAACAGCTGGAGCGAAAGATCCACTATCGCCAGCAGGATCTGGTGGACTACTCGCCGGTCAGCGAAAAACACCTTGCCGACGGCATGACGGTCGGCGAACTCTGTGCCGCCGCCATTACCATGAGCGATAACAGCGCCGCCAATCTGCTGCTGGCCACCGTCGGCGGCCCCGCAGGATTGACTGCCTTTTTGCGCCAGATCGGCGACAACGTCACCCGCCTTGACCGCTGGGAAACGGAACTGAATGAGGCGCTTCCCGGCGACGCCCGCGACACCACTACCCCGGCCAGCATGGCCGCGACCCTGCGCAAGCTGCTGACCAGCCAGCGTCTGAGCGCCCGTTCGCAACGGCAGCTGCTGCAGTGGATGGTGGACGATCGGGTCGCCGGACCGTTGATCCGCTCCGTGCTGCCGGCGGGCTGGTTTATCGCCGATAAGACCGGAGCTGGCGAACGGGGTGCGCGCGGGATTGTCGCCCTGCTTGGCCCGAATAACAAAGCAGAGCGCATTGTGGTGATTTATCTGCGGGATACCCCGGCGAGCATGGCCGAGCGAAATCAGCAAATCGCCGGGATCGGCGCGGCGCTGATCGAGCACTGGCAACGCTAA

>blaSHV-33

ATGCGTTATATTCGCCTGTGTATTATCTCCCTGTTAGCCACCCTGCCGCTGGCGGTACACGCCAGCCCGCAGCCGCTTGAGCAAATTAAACTAAGCGAAAGCCAGCTGTCGGGCCGCGTAGGCATGATAGAAATGGATCTGGCCAGCGGCCGCACGCTGACCGCCTGGCGCGCCGATGAACGCTTTCCCATGATGAGCACCTTTAAAGTAGTGCTCTGCGGCGCAGTGCTGGCGCGGGTGGATGCCGGTGACGAACAGCTGGAGCGAAAGATCCACTATCGCCAGCAGGATCTGGTGGACTACTCGCCGGTCAGCGAAAAACACCTTGCCGACGGCATGACGGTCGGCGAACTCTGTGCCGCCGCCATTACCATGAGCGATAACAGCGCCGCCAATCTGCTGCTGGCCACCGTCGGCGGCCCCGCAGGATTGACTGCCTTTTTGCGCCAGATCGGCGACAACGTCACCCGCCTTGACCGCTGGGAAACGGAACTGAATGAGGCGCTTCCCGGCGACGCCCGCGACACCACTACCCCGGCCAGCATGGCCGCGACCCTGCGCAAGCTGCTGACCAGCCAGCGTCTGAGCGCCCGTTCGCAACGGCAGCTGCTGCAGTGGATGGTGGACGATCGGGTCGCCGGACCGTTGATCCGCTCCGTGCTGTCGGCGGGCTGGTTTATCGCCGATAAGACCGGAGCTGGCGAACGGGGTGCGCGCGGGATTGTCGCCCTGCTTGGCCCGAATAACAAAGCAGAGCGCATTGTGGTGATTTATCTGCGGGATACCCCGGCGAGCATGGCCGAGCGAAATCAGCAAATCGCCGGGATCGGCGCGGCGCTGATCGAGCACTGGCAACGCTAA
