# Supplementary material for: Piperacillin-tazobactam resistance in Klebsiella pneumoniae is often associated with IS26-mediated blaSHV-1 amplification in a widespread Klebsiella-adapted plasmid
Source: Antimicrob Agents Chemother. 2026 Mar 24;70(5):e01682-25. doi: 10.1128/aac.01682-25 (PMC13148056; doi:10.1128/aac.01682-25)
Supplement: Supplemental figures — Fig. S1 to S6. [file aac.01682-25-s0002.pdf]

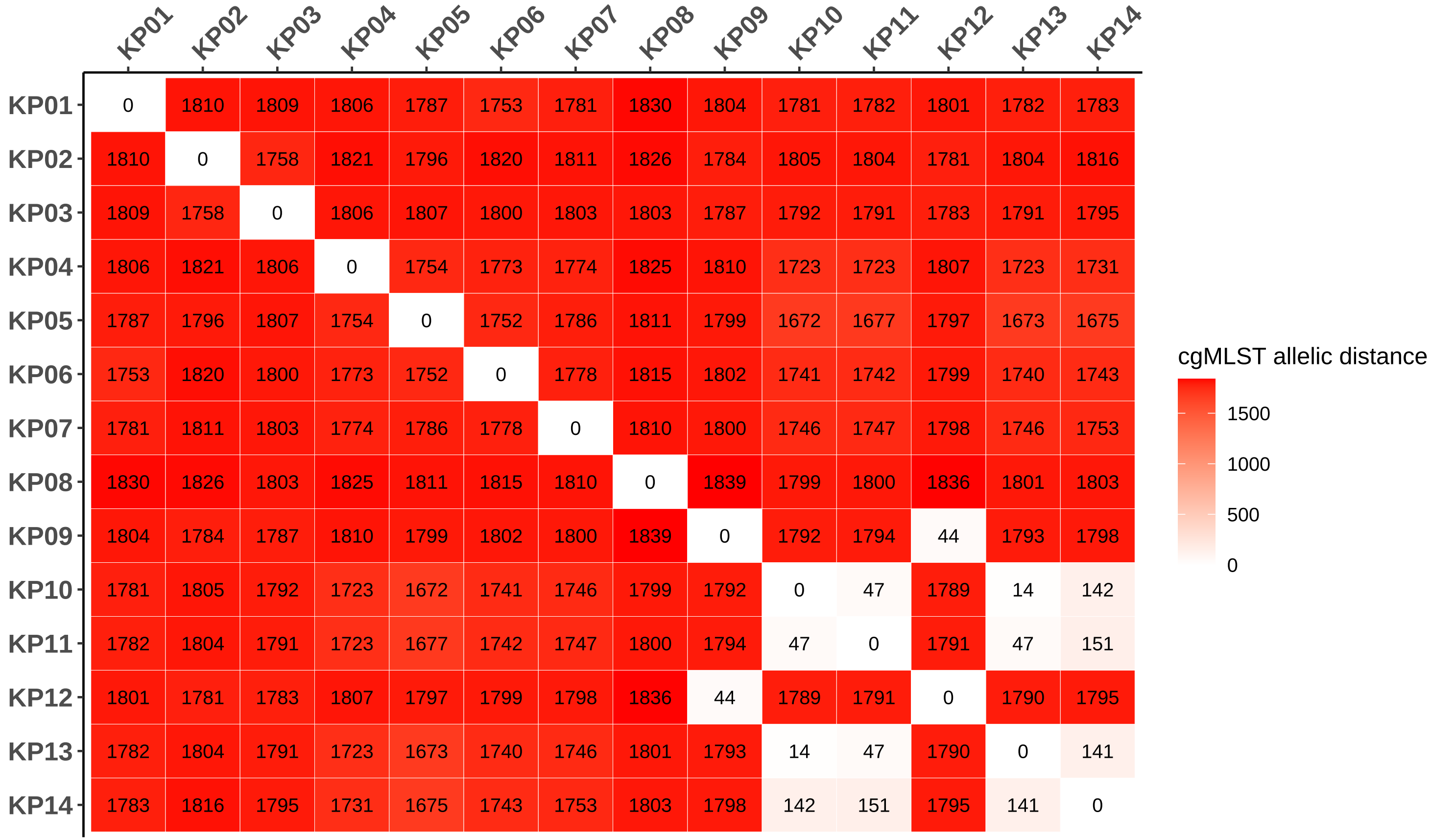

	KP01	KP02	KP03	KP04	KP05	KP06	KP07	KP08	KP09	KP10	KP11	KP12	KP13	KP14
KP01	0	1810	1809	1806	1787	1753	1781	1830	1804	1781	1782	1801	1782	1783
KP02	1810	0	1758	1821	1796	1820	1811	1826	1784	1805	1804	1781	1804	1816
KP03	1809	1758	0	1806	1807	1800	1803	1803	1787	1792	1791	1783	1791	1795
KP04	1806	1821	1806	0	1754	1773	1774	1825	1810	1723	1723	1807	1723	1731
KP05	1787	1796	1807	1754	0	1752	1786	1811	1799	1672	1677	1797	1673	1675
KP06	1753	1820	1800	1773	1752	0	1778	1815	1802	1741	1742	1799	1740	1743
KP07	1781	1811	1803	1774	1786	1778	0	1810	1800	1746	1747	1798	1746	1753
KP08	1830	1826	1803	1825	1811	1815	1810	0	1839	1799	1800	1836	1801	1803
KP09	1804	1784	1787	1810	1799	1802	1800	1839	0	1792	1794	44	1793	1798
KP10	1781	1805	1792	1723	1672	1741	1746	1799	1792	0	47	1789	14	142
KP11	1782	1804	1791	1723	1677	1742	1747	1800	1794	47	0	1791	47	151
KP12	1801	1781	1783	1807	1797	1799	1798	1836	44	1789	1791	0	1790	1795
KP13	1782	1804	1791	1723	1673	1740	1746	1801	1793	14	47	1790	0	141
KP14	1783	1816	1795	1731	1675	1743	1753	1803	1798	142	151	1795	141	0

Figure S1: Core genome MLST-based (cgMLST) genetic distances between strains. Genetic distances were computed from an all-vs-all genome comparison using the *K. pneumoniae/variicola/quasipneumoniae* cgMLST scheme (https://www.cgmlst.org/ncs/schema/Kpneumoniae985/), which consists of 2,358 loci.

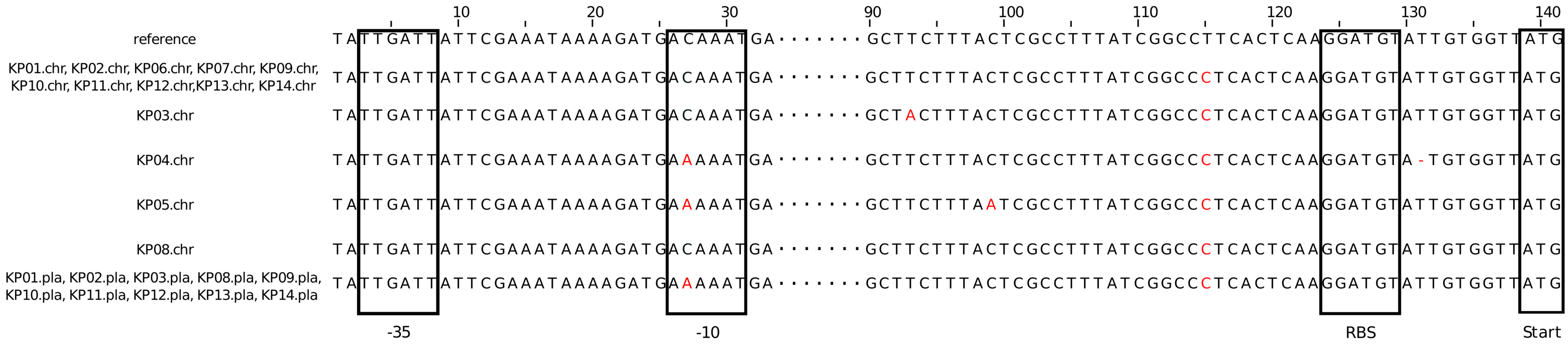

Figure S2: Nucleotide alignment of the *bla*$_{SHV}$ promoter among the studied strains. The sequence of *K. pneumoniae* HS11286 was used as reference. The start codon, ribosome binding site (RBS), and the -10 and -35 boxes are outlined in black. Nucleotide variations compared to the reference are highlighted in red. The location of *bla*$_{SHV}$ is indicated as "chr" for chromosomal or "pla" for plasmid.

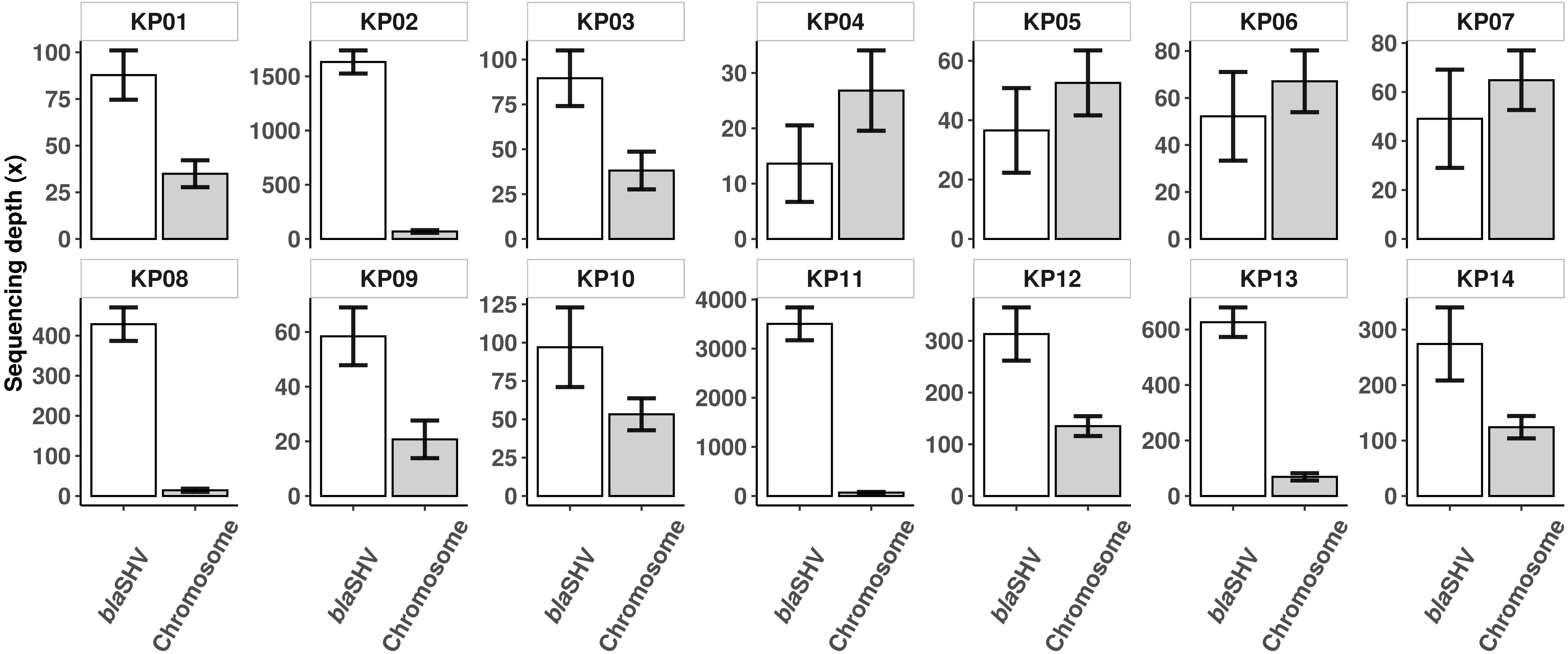

Figure S3: Sequencing depth of *bla*$_{SHV}$ and the chromosome. For each strain, Illumina short reads were mapped to the *bla*$_{SHV-1v1}$ gene and MLST genes to determine the sequencing depth of *bla*$_{SHV}$ and the chromosome, respectively. Bar plots represent the mean depth values, and error bars indicate the standard deviation.

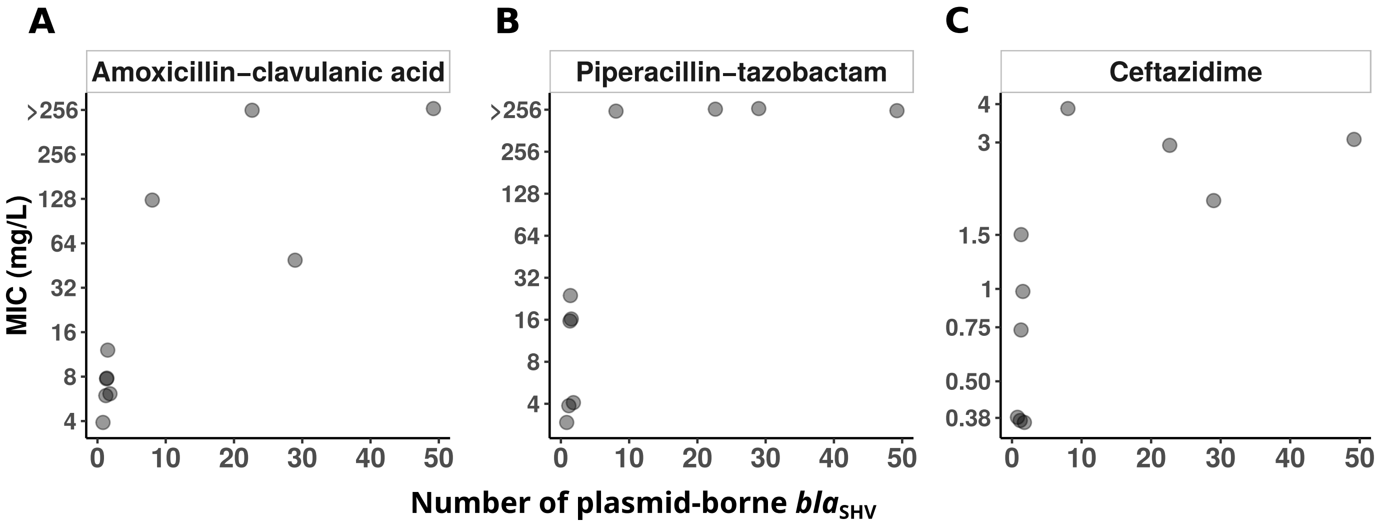

Figure S4: Number of plasmid-borne *bla*$_{\text{SHV-1}}$ copies and MICs of (A) amoxicillin-clavulanic acid, (B) piperacillin-tazobactam, and (C) ceftazidime. MICs were determined using E-tests (BioMérieux) for amoxicillin-clavulanic acid and ceftazidime, and MIC Test Strips (Liofilchem) for piperacillin-tazobactam. Each point represents a strain, and only strains carrying a plasmid-borne copy of *bla*$_{\text{SHV-1}}$ are shown (KP01, KP02, KP03, KP08, KP09, KP10, KP11, KP12, KP13, KP14).

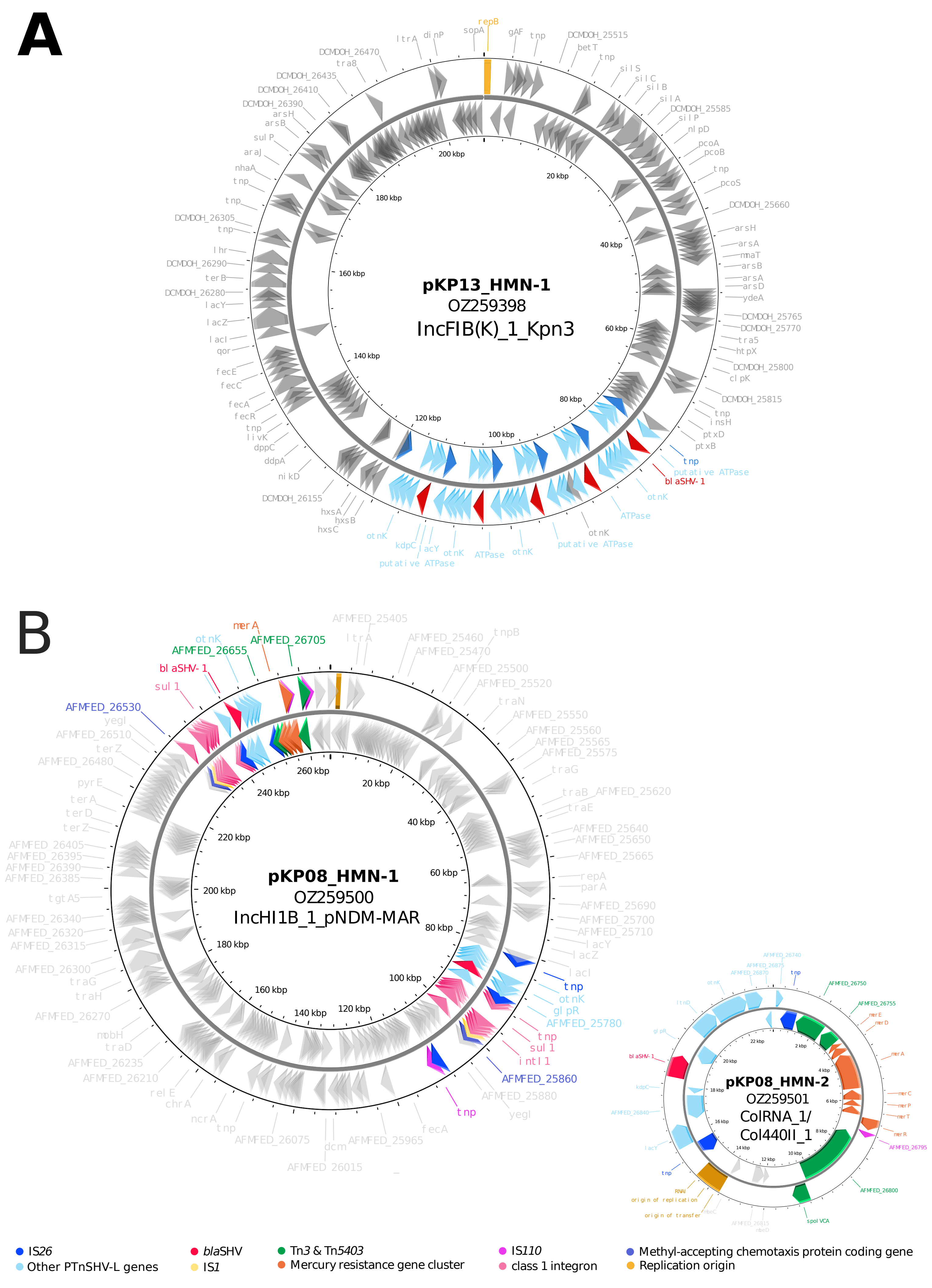

Figure S5: Plasmids carrying bla_{SHV-1} copies in KP08 and KP13. (A) Physical map of pKP13-HMN-1 from KP13 strain. (B) Physical maps of pKP08-HMN-1 and pKP08_HMN-2 from KP08 strain. Colored arrows show different types of genes, as indicated in the key.

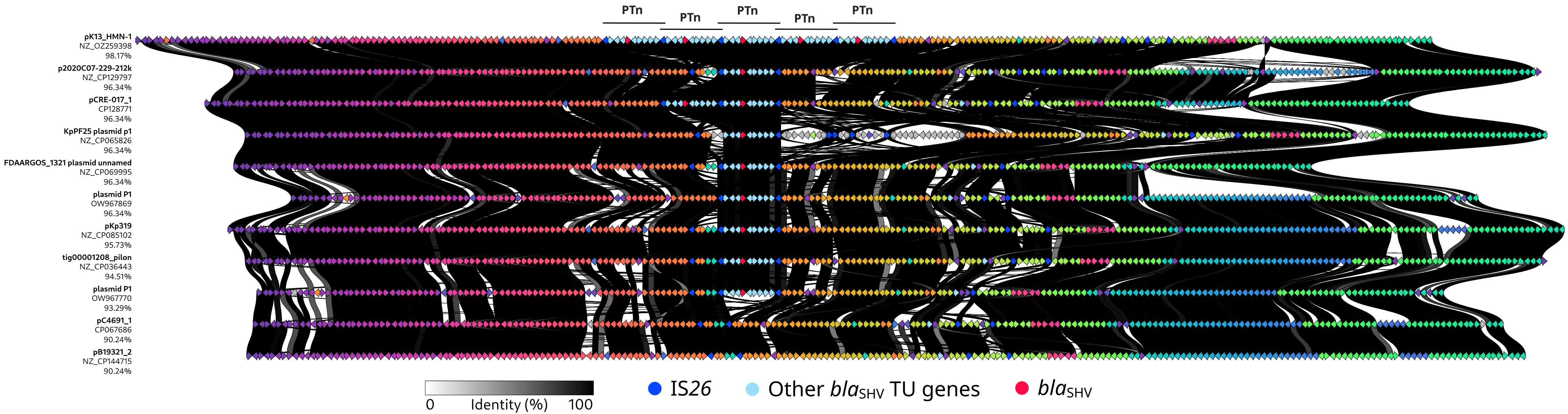

Figure S6: Alignment of pKP13-HMN-1 and fully assembled plasmids from the PLSDB plasmid database that carry PTnSHV-L with *bla*$_{SHV-1v1}$. Colored arrows show different types of genes, as indicated in the key. Shading between diagrams indicates the level of protein identity (minimum 60%). The PTnSHV-L copies of pKP13-HMN-1 are highlighted with a black line on the top of the map. Plasmid names, accession numbers, and the percentage of pKP13-HMN-1 genes identified are indicated on the left side of each map.
